# Enhancement of Electromagnetic Wave Shielding Effectiveness of Carbon Fibers via Chemical Composition Transformation Using H_2_ Plasma Treatment

**DOI:** 10.3390/nano10081611

**Published:** 2020-08-17

**Authors:** Hyun-Ji Kim, Gi-Hwan Kang, Sung-Hoon Kim, Sangmoon Park

**Affiliations:** Department of Energy and Chemical Engineering, Silla University, Busan 46958, Korea; khyunj32@gmail.com (H.-J.K.); nice_gyan@naver.com (G.-H.K.); spark@silla.ac.kr (S.P.)

**Keywords:** electromagnetic wave shielding effectiveness, carbon-based nonwoven fabrics, H_2_ plasma treatment, absorption loss shielding mechanism, chemical composition transformation

## Abstract

H_2_ plasma treatment was performed on carbon-based nonwoven fabrics (c-NFs) in a 900 W microwave plasma-enhanced chemical vapor deposition system at 750 °C and 40 Torr. Consequently, the electromagnetic wave shielding effectiveness (SE) of the c-NFs was significantly enhanced across the operating frequency range of 0.04 to 20.0 GHz. We compared the electromagnetic wave SE of the H_2_ plasma-treated c-NFs samples with that of native c-NFs samples coated with nano-sized Ag particles. Despite having a lower surface electrical conductivity, H_2_ plasma-treated c-NFs samples exhibited a considerably higher electromagnetic wave SE than the Ag-coated c-NFs samples, across the relatively high operating frequency range of 7.0 to 20.0 GHz. The carbon component of H_2_ plasma-treated c-NFs samples increased significantly compared with the oxygen component. The H_2_ plasma treatment transformed the alcohol-type (C–O–H) compounds formed by carbon-oxygen bonds on the surface of the native c-NFs samples into ether-type (C–O–C) compounds. On the basis of these results, we proposed a mechanism to explain the electromagnetic wave SE enhancement observed in H_2_ plasma-treated c-NFs.

## 1. Introduction

Electromagnetic waves are composed of oscillating electric and magnetic fields. Therefore, materials with electromagnetic wave shielding capabilities are expected to interact with either one or both of these fields. Shielding of electromagnetic waves can occur via either reflection loss, absorption loss, or multiple reflection loss [1,2,3,4]. Reflection loss and absorption loss are considered the main shielding mechanisms for achieving efficient absorption loss greater than 10 dB [5,6,7,8]. The shielding effectiveness (SE) of electromagnetic wave shielding materials is given in units of dB and can be estimated using the following empirical equation, which was proposed by Simon [8]: SE = 50 + 10log(*ρf*)^−1^ + 1.7*t*(*f*/*ρ*)^1/2^, where *ρ* is resistivity, *t* is the thickness of the shielding material, and *f* is the operating frequency. In this equation, reflection loss, that is, 10log(*ρf*)^−1^, decreases with increasing operating frequency. However, absorption loss, that is, 1.7*t*(*f*/*ρ*)^1/2^, increases with increasing operating frequency. Therefore, at high operating frequencies required by technologies such as fifth-generation wireless networks, the absorption loss characteristics of shielding materials are especially important for effectively protecting electromagnetic waves.

Carbon microcoils used as electromagnetic wave shielding materials have a helical structure, with the individual carbon nanofibers oriented along the growth direction of the microcoil axis [9,10]. When an incoming electromagnetic wave reaches the carbon microcoils, the electric current flows into the individual helical-type carbon nanofibers in varying directions, thereby inducing an electromotive force and generating a variable magnetic field [11,12]. The geometry of the carbon microcoils stops and rotates the incoming electromagnetic wave within the generated variable magnetic field. Thus, the incoming electromagnetic wave energy is absorbed into the carbon microcoils and is converted into thermal energy [13].

Similar to carbon microcoils, carbon-based nonwoven fabrics (c-NFs) consist of randomly oriented carbon fibers. When an incoming electromagnetic wave reaches these fibers, the direction of the flowing electric current may vary, as in the case of helical-type carbon microcoils. Subsequently, c-NFs induce an electromotive force, eventually generating a variable magnetic field. Accordingly, c-NFs absorb the electromagnetic waves, thus shielding against such waves. In our previous work, we reported the superior absorption characteristics and SE of c-NFs [14].

Reflection loss of electromagnetic radiation in a material occurs owing to the interaction between the electric field of the electromagnetic radiation and the material. However, absorption loss in a material occurs as a result of interactions between both the electric and magnetic fields of the electromagnetic radiation and the material. The penetration of electromagnetic waves within a limited depth range below the surface of a shielding material is known as the skin effect. This effect is noticeable at high operating frequencies [2,5]. The skin depth (*δ*) is defined as the depth at which the electromagnetic wave field inside the material falls to 1/e of its incident value; it can be calculated using the following equation: *δ* = (*πσfμ*)^−1/2^ [2,5], where *σ*, *f*, and *μ* denote the electrical conductivity, operating frequency, and magnetic permeability, respectively. This equation shows that skin depth decreases with increasing frequency, electrical conductivity, and magnetic permeability. Therefore, an electromagnetic wave shielding material should have a high electrical conductivity as well as a high magnetic permeability at high operating frequencies.

In the present work, we attempted to improve the electromagnetic wave SE of carbon-based materials by increasing the electrical conductivities of well-made c-NFs with a variable magnetic field. To achieve this, we developed a simple pretreatment method involving H_2_ plasma treatment or Ag coating to enhance the electrical conductivity of the c-NFs samples. The morphologies, electrical conductivities with and without pretreatment, and variations in chemical composition of the c-NFs samples were investigated and discussed.

## 2. Materials and Methods

**Figure 1:**
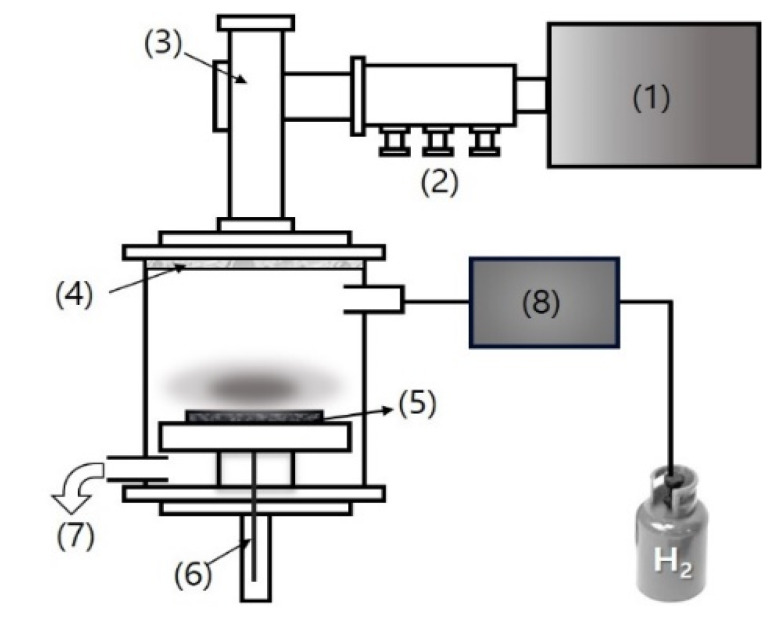
Schematic of the microwave plasma-enhanced chemical vapor deposition (MPECVD) system: (1) microwave remote power head; (2) three-stub tuner; (3) plasma applicator; (4) microwave window; (5) graphite substrate; (6) manipulating heater stage; (7) pumping system; and (8) mass flow controller system.

We fabricated c-NFs using a modified carding machine [15]. H_2_ plasma treatment was performed on the c-NFs samples using a microwave plasma-enhanced chemical vapor deposition (MPECVD) system. In general, the experimental parameters for plasma processing using MPECVD systems are microwave generator power, substrate temperature, and plasma reaction time [16,17]. Occasionally, total pressure is also considered. The total pressure condition is typically set in the pre-experimental phase to generate the plasma. Therefore, in this work, the total pressure is initially set in the reactor to form the plasma. We performed several experiments as functions of the microwave generator power, substrate temperature, and plasma reaction time to determine the optimal conditions for the H_2_ plasma treatment process. In the end, we determined that H_2_ plasma treatment for 5 min was optimal for investigating the mechanism that caused the SE enhancement. Figure 1 shows the MPECVD system, and Table 1 lists the detailed experimental conditions under which the H_2_ plasma treatment was performed.

**Figure 2:**
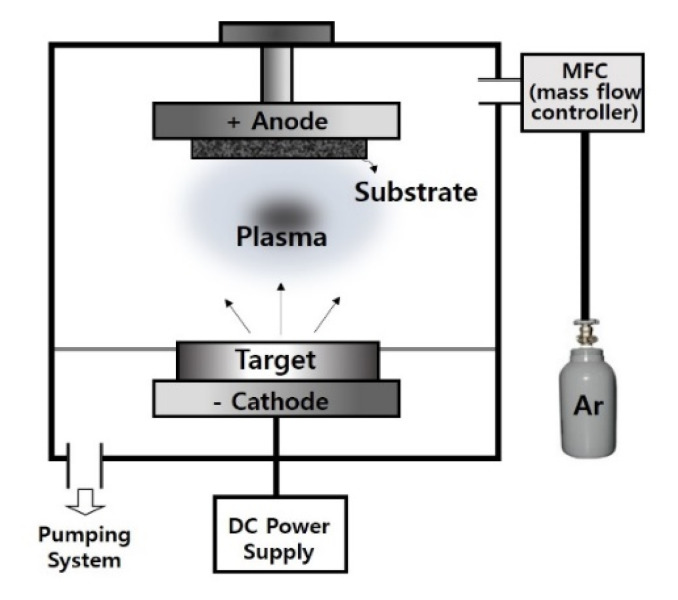
Schematic diagram of the direct current (DC) sputtering system used to produce Ag-coated c-NFs.

Ag coatings of c-NFs were performed using a home-made direct current (DC) sputtering system. Figure 2 shows the system, and Table 2 lists the experimental conditions for the Ag coating process.

**Figure 3:**
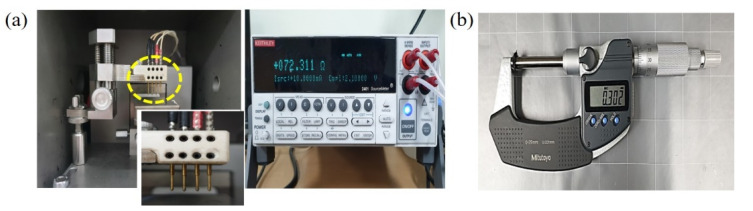
(**a**) Optical images of the four-point probe (left) and source meter (right) used in the experiments; (**b**) optical image of the micrometer used to measure the thickness of the samples.

The morphologies of the samples were investigated in detail using a field-emission scanning electron microscopy (FESEM; S-4200, Tokyo, Japan), while the chemical compositions of each sample were examined using an energy dispersive X-ray spectroscopy (EDS; JSM-6700F, Tokyo, Japan). Furthermore, composition analysis of the sample surfaces was performed using an X-ray photoelectron spectroscopy (XPS; Theta Probe, Waltham, MA, USA) with a spot size of 400 μm. Resistivity values were obtained using a four-point probe (labsysstc-400, Busan, Republic of Korea) connected to a source meter (2400 Source Meter, Cleveland, OH, USA) and by performing calculations using Ohm’s law with a correction factor (see Figure 3a) [3,18,19]. The following is the process for the electrical conductivity measurement in this work. The electrical resistivity of the samples was measured using the four-point probe system, according to the method proposed by Smits [18]. The four-point probe system consists of four straight-lined probes with equal inter-probe spacing of 3.0 mm. A constant current (*I*) was supplied through the two outer probes. Using the two inner probes, we measured the output voltage (*V*) [3]. Furthermore, the correction factors (*C* and *F*) were obtained from Smits’s study [18]. Surface and volume resistivity were calculated using the following equation [3,18]:

(1) $$\mathrm{Surface}\;\mathrm{resistivity}:\;\rho_{s}\;=\;\frac{V}{I}C\left(\frac{a}{d},\frac{d}{s}\right)\;\mathrm{Volume}\;\mathrm{resistivity}:\;\rho_{v}\;=\;\rho_{s}wF(\frac{w}{s})$$

where *a*, *d*, *w,* and *s* denote the length, width, and thickness of the sample and an inter-probe spacing, respectively.

The thicknesses of the samples were measured using a micrometer (406-250-30, Nakagawa, Japan), as shown in Figure 3b. However, the thickness difference of the samples was not considered in this work owing to the short time (5 min) of the H_2_ plasma treatment process and/or the small-sized (60 nm in diameter) Ag particle coating treatment process performed on the carbon fibers of the samples.

**Figure 4:**
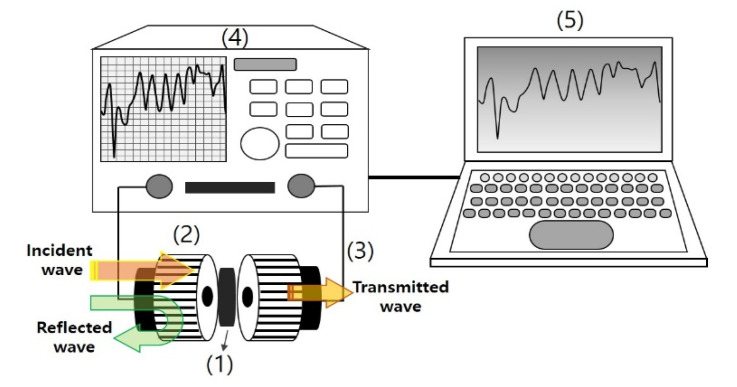
Schematic of the vector network analyzer (VNA) system: (1) sample; (2) wave-guide test holders; (3) coaxial cables; (4) VNA; and (5) computer.

The SE values of the c-NFs samples were measured using the waveguide method with a vector network analyzer (VNA; 37369C, Kanagawa, Japan), as shown in Figure 4. The setup consisted of a sample holder with its exterior connected to the VNA system (Figure 4).

A coaxial sample holder and coaxial transmission test specimen were set up according to the waveguide method. We measured scattering parameters (*S_11_* and *S_21_*) in the 0.04 to 20.0 GHz frequency range using a VNA [20,21,22,23]. The power coefficients, namely, reflectivity (*R*), absorptivity (*A*), and transmissivity (*T*), were calculated using the equations *R* = *P_R_*/*P_I_* =|*S_11_*|^2^ and *T* = *P_T_*/*P*_I_ = |*S_21_*|^2^, where *P_I_*, *P_R_*, *P_A_*, and *P_T_* are the incident, reflected, absorbed, and transmitted powers of an electromagnetic wave, respectively. The power coefficient relationship is expressed as *R* + *A* + *T* = 1. The electromagnetic wave SE was calculated from the scattering parameters using the following equations:

*SE_tot_* = −10log *T*

*SE_R_* = −10log (1 − *R*)

*SE_A_* = −10log [*T*/(1 − *R*)]

where *SE_to_*_t_, *SE_R_*, and *SE_A_* denote the total, reflection, and absorption SE values, respectively [23].

## 3. Results

**Figure 5:**
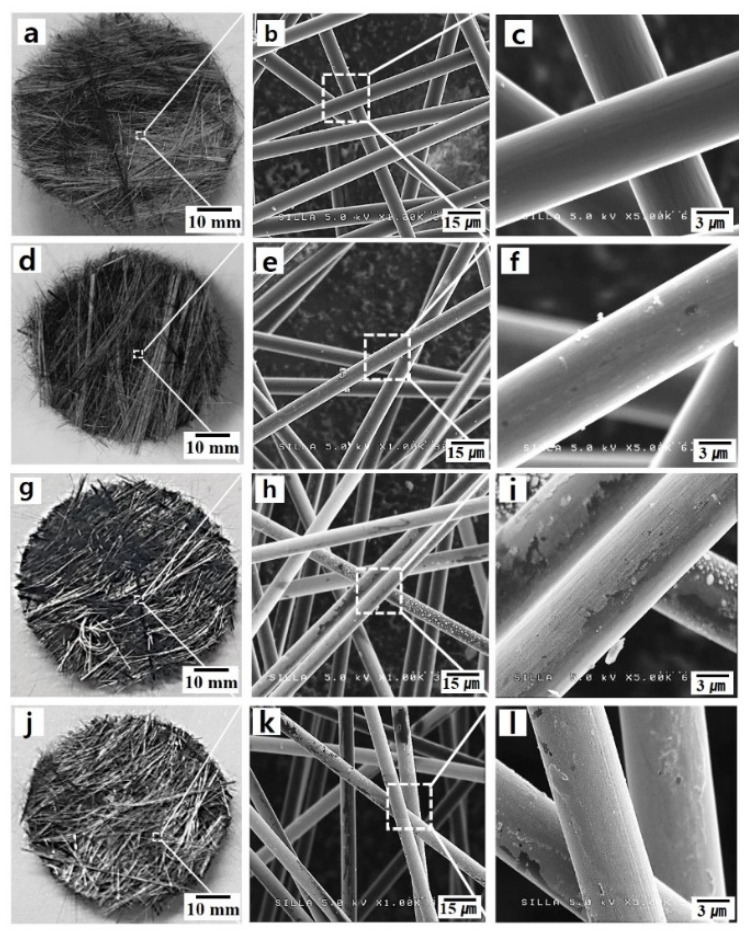
(**a**), (**d**), (**g**), and (**j**) are photographs; (**b**), (**e**), (**h**), and (**k**) are field-emission scanning electron microscopy (FESEM) images; and (**c**), (**f**), (**i**), and (**l**) are magnified FESEM images. From top of bottom, each column of images shows native carbon-based nonwoven fabrics (c-NFs) samples, plasma-treated c-NFs samples, Ag-coated c-NFs samples, and plasma-treated Ag-coated c-NFs samples, respectively.

**Figure 6:**
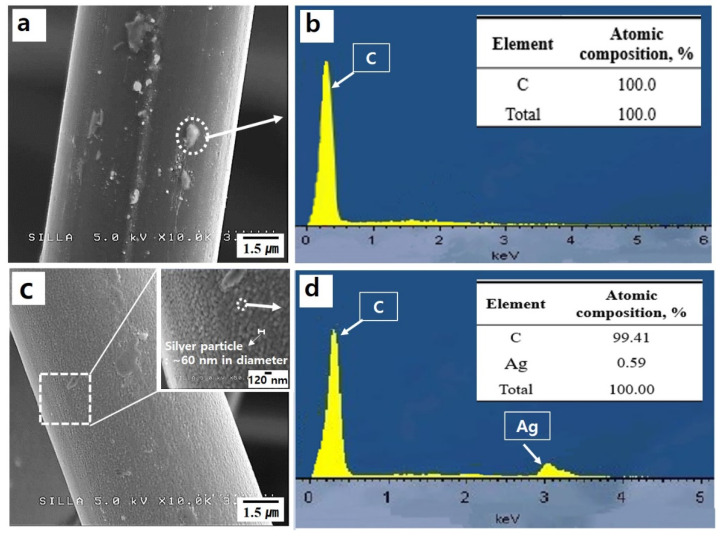
(**a**) A magnified FESEM image of an individual carbon fiber in a plasma-treated c-NFs sample and (**b**) the corresponding energy dispersive X-ray spectroscopy (EDS) spectrum obtained for the area inside the dotted-circle in Figure 6a. (**c**) A magnified FESEM image of an individual carbon fiber has inset showing silver particles in an Ag-coated c-NFs sample; and (**d**) the corresponding EDS spectrum for the area inside the dotted-circle in the inset of Figure 6c.

Figure 5 shows photographs and FESEM images of native c-NFs samples, plasma-treated c-NFs samples, Ag-coated c-NFs samples, and plasma-treated Ag-coated c-NFs samples. As shown in Figure 5b, the native c-NFs samples contained numerous intersection points of randomly oriented individual carbon fibers. When an incoming electromagnetic wave reaches an individual carbon fiber, the electric current from the electromagnetic wave flows in a different direction from the point of intersection. Consequently, the direction of the flowing electric current varies as it would for helical-type carbon microcoils. Therefore, native c-NFs can induce an electromotive force, eventually producing a variable magnetic field, and thereby enhancing the magnetic properties and electromagnetic wave SE of the c-NFs. Consequently, the electromagnetic wave SE of a native c-NFs is enhanced via the absorption mechanism of an electromagnetic wave. The individual carbon fibers that constitute native c-NFs have clean surfaces (see Figure 5c), while the fibers of c-NFs that have been plasma-treated for 5 min have submicron-sized particles attached to their surfaces, as shown in Figure 5f and Figure 6a.

The EDS spectrum in Figure 6b shows that these attached submicron-sized particles are composed mainly of carbon. The individual carbon fibers that constitute Ag-coated c-NFs have stained surfaces, as shown in Figure 5i. The highly magnified FESEM images of the Ag-coated c-NFs samples in Figure 6 reveal the presence of numerous nano-sized Ag particles on the surface of the individual carbon fibers. The average Ag particle diameter was approximately 60 nm. The individual carbon fibers that constituted the Ag-coated c-NFs samples also treated with H_2_ plasma had surface morphologies similar to those of the Ag-coated c-NFs samples.

The sheet resistance (*R_s_*) values of the native c-NFs samples, plasma-treated c-NFs samples, Ag-coated c-NFs samples, and plasma-treated Ag-coated c-NFs samples were measured using a four-point probe. All of the samples appeared to have a thickness of 1.5 mm. As shown in Table 3, the electrical conductivity of the plasma-treated c-NFs samples was higher than that of the native c-NFs samples, while the electrical conductivity of the Ag-coated c-NFs samples was higher than those of both the plasma-treated and native samples.

**Figure 7:**
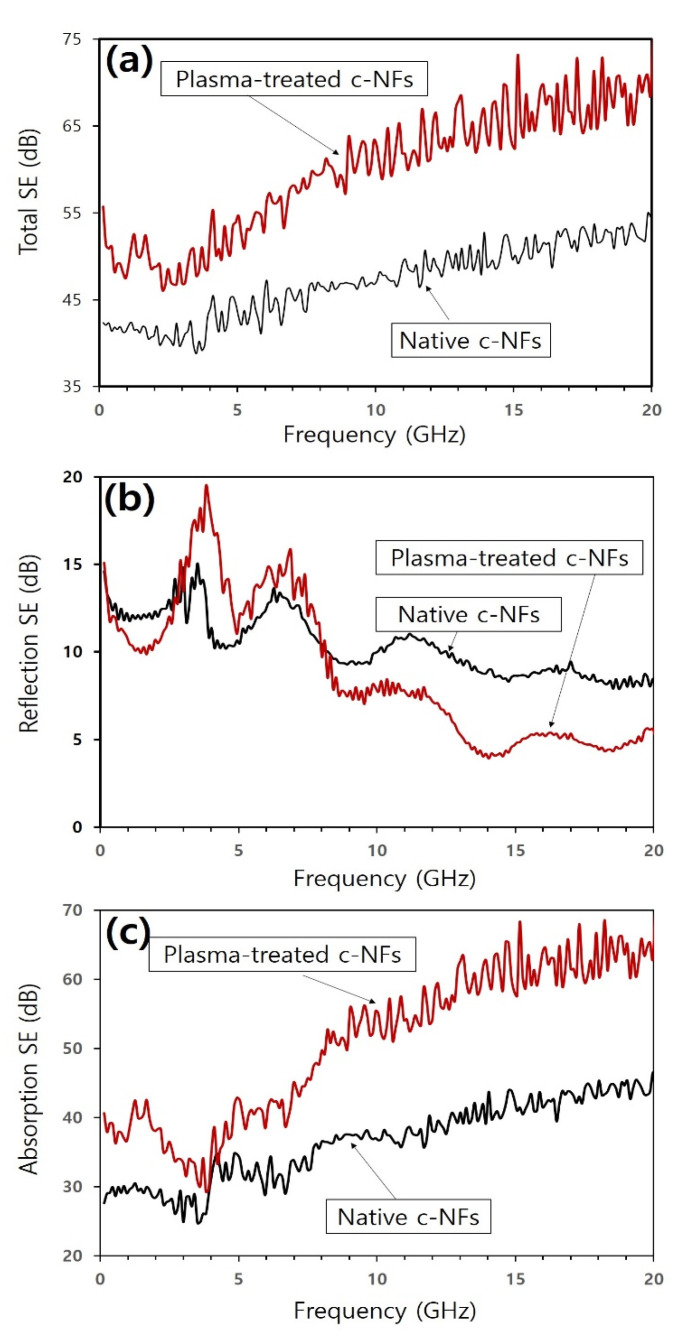
(**a**) Total shielding effectiveness (SE) spectra; (**b**) components of SE spectra owing to reflection; and (**c**) components of SE spectra owing to absorption for both native and plasma-treated c-NFs samples.

The total SE values of the plasma-treated c-NFs samples were considerably higher than those of the native c-NFs samples across the entire range of operating frequencies (see Figure 7a). Figure 7b,c show the SE values due to reflection loss and absorption loss, respectively, of the native and plasma-treated c-NFs samples. As shown in Figure 7b, the reflection loss SE values of the plasma-treated c-NFs samples were lower than those of the native c-NFs samples for operating frequencies between 0.04 and 3.0 GHz. However, the values were higher for operating frequencies between 3.0 and 8.0 GHz before finally lowering in the 8.0 to 20 GHz range. As shown in Figure 7c, for the absorption loss, the SE values of plasma-treated c-NFs were greater than those of the native c-NFs throughout the range of operating frequencies. These results strongly indicate that the increase in total SE for plasma-treated c-NFs samples when compared with native c-NFs samples was mainly owing to enhanced absorption loss throughout the range of studied operating frequencies.

**Figure 8:**
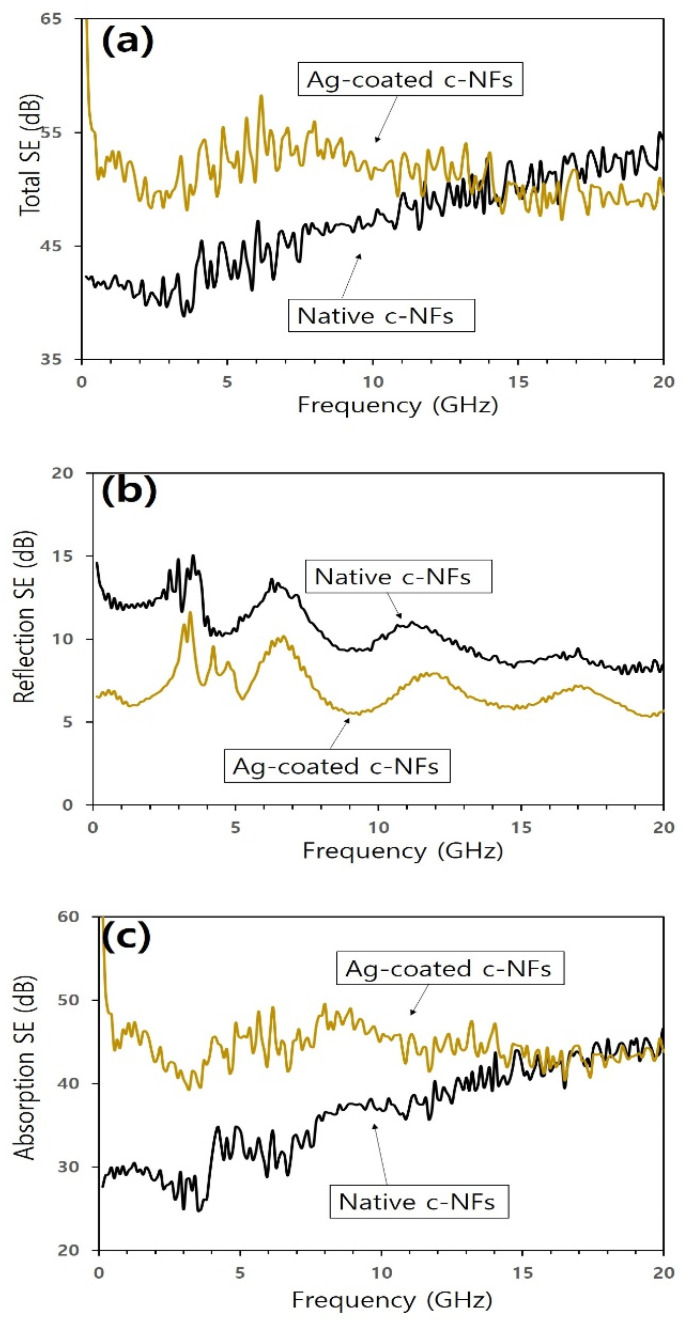
(**a**) Total SE spectra; (**b**) components of SE spectra owing to reflection; and (**c**) components of SE spectra owing to absorption for both native and Ag-coated c-NFs samples.

The total SE values obtained for Ag-coated c-NFs samples were considerably higher than those obtained for native c-NFs samples in the range of operating frequencies from 0.04 to 10.0 GHz (see Figure 8a). However, the difference in total SE values between the samples gradually decreased as the operating frequency increased from 8.0 to 12.0 GHz. Between 12.0 and 16.0 GHz, the samples exhibited similar total SE values, but above 16.0 GHz, the total SE of the Ag-coated c-NFs samples was slightly lower than that of the native c-NFs samples. These results indicate that the SE enhancement achieved through Ag coating occurred over a relatively low range of operating frequencies. Figure 8b,c show the components of the SE spectra owing to reflection loss and absorption loss, respectively, for native c-NFs samples and Ag-coated c-NFs samples. As shown in the reflection loss spectra, the SE of the Ag-coated c-NFs samples was lower than that of the native c-NFs samples throughout the range of operating frequencies (see Figure 8b). As shown in the absorption loss spectra, the SE of the Ag-coated c-NFs samples was higher than that of the native c-NFs samples in the range of 0.04 to 8.0 GHz. However, the difference between the SE values obtained for the Ag-coated c-NFs samples and those obtained for the native c-NFs samples gradually decreased as the operating frequency increased from 8.0 to 16.0 GHz. Above 16.0 GHz, similar values were obtained for both samples. These results indicate that the increased SE values obtained for Ag-coated c-NFs samples can be attributed to enhanced absorption loss at low operating frequencies.

**Figure 9:**
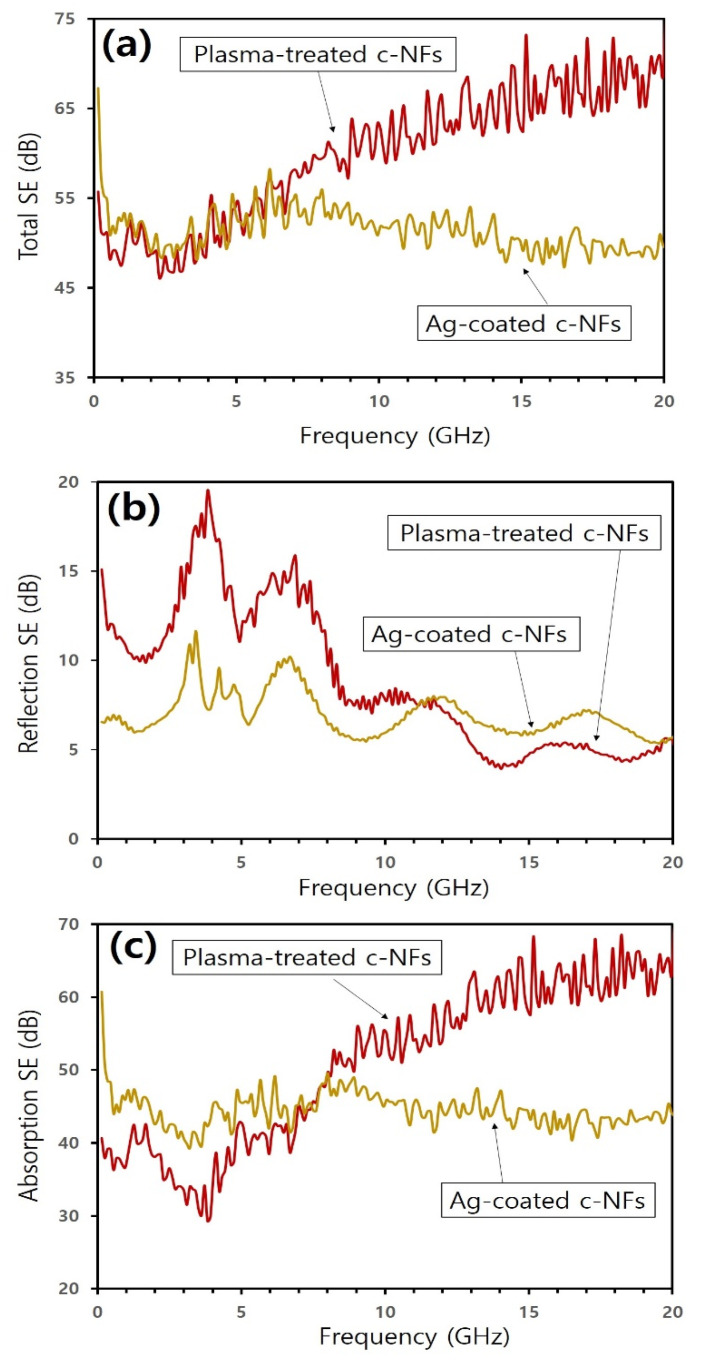
(**a**) Total SE spectra; (**b**) components of SE spectra owing to reflection; and (**c**) components of SE spectra owing to absorption for both plasma-treated and Ag-coated c-NFs samples.

Figure 9a–c show the total SE spectra, reflection loss spectra, and absorption loss spectra, respectively, for plasma-treated c-NFs samples and Ag-coated c-NFs samples. In the 0.04 to 7.0 GHz operating frequency range, the total SE values obtained for the Ag-coated samples were similar to those obtained for the plasma-treated samples. Above 7.0 GHz, however, the total SE of the Ag-coated samples decreased slightly, while that of the plasma-treated samples increased. Consequently, the total SE values obtained for the plasma-treated samples were higher than those obtained for the Ag-coated samples in the 7.0 to 20.0 GHz operating frequency range. As shown in the reflection loss spectra in Figure 9b, the SE values obtained for the plasma-treated samples were higher than those obtained for the Ag-coated samples for operating frequencies between 0.04 and 11.0 GHz, but lower for frequencies above 12.0 GHz. As shown in the absorption loss spectra in Figure 9c, the SE values obtained for the plasma-treated samples were lower than those obtained for the Ag-coated samples for frequencies between 0.04 and 8.0 GHz, but higher for frequencies between 8.0 and 20.0 GHz. Furthermore, the difference between the values obtained for these samples gradually increased as the operating frequency increased. Therefore, the enhanced total SE exhibited by the plasma-treated samples when compared with the Ag-coated samples could be attributed primarily to enhanced absorption loss at high operating frequencies between 7.0 and 20.0 GHz.

The combined results contained in Figure 7, Figure 8 and Figure 9 indicate that subjecting native c-NFs samples to either H_2_ plasma treatment or Ag coating enhanced the total SE of the samples for low operating frequencies. As shown in Table 3, the enhanced total SE of the treated samples can be attributed to the fact that the treated samples exhibited higher electrical conductivity than the native samples.

The data in Figure 9 reveal that H_2_ plasma treatment enhanced electromagnetic wave SE more significantly than Ag coating. This was owing to an enhancement in the absorption loss effects at high operating frequencies above 7.0 GHz and occurred even though the surface electrical conductivity of the H_2_ plasma-treated samples was lower than that of the Ag-coated samples. Skin depth (*δ*) decreases as electrical conductivity (*σ*) and magnetic permeability (*μ*) increase [2,8]. Therefore, the materials used to shield electromagnetic wave radiation should have a high electrical conductivity as well as a high magnetic permeability. The obtained results indicate that H_2_ plasma treatment enhances the magnetic characteristics of native c-NFs to a greater extent than Ag coating. Furthermore, the results also indicate that H_2_ plasma treatment may alter the surface chemical composition of native c-NFs.

**Figure 10:**
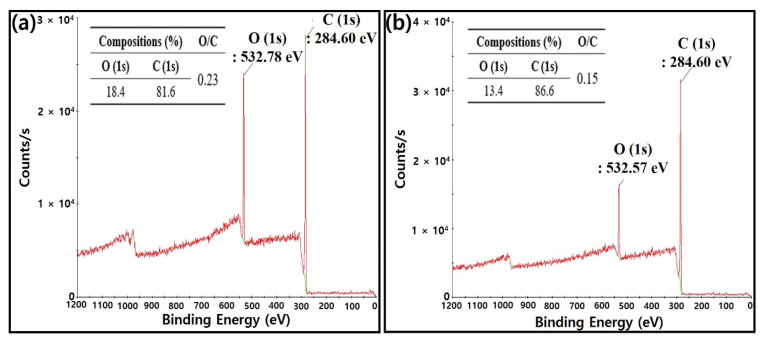
X-ray photoelectron spectroscopy (XPS) spectra and compositions of (**a**) native c-NFs and (**b**) plasma-treated c-NFs.

To investigate plasma treatment-induced variations in the surface chemical composition of native c-NFs, we performed XPS analysis on both native c-NFs samples and plasma-treated c-NFs samples, with the results shown in Figure 10. Table 4 lists the elemental compositions and binding energies studied via XPS.

Oxygen and carbon components are typically observed on the surface of the carbon fibers that constitute native c-NFs, as shown in Figure 10 [24,25]. The presence of a native oxygen component on the surface of a carbon fiber is owing to the residual oxygen that remains in the reactor during the reaction. A comparison of the data in Figure 10a,b revealed a notable change in the chemical composition of the carbon fiber surface after treatment with H_2_ plasma for only 5 min. Specifically, the plasma treatment increased the carbon component of the surface composition by 5%. Table 4 lists the O (1s) binding energy values obtained for samples of native c-NFs and plasma-treated c-NFs. The O (1s) binding energy values of the native c-NFs samples corresponded to alcohol-type compounds (C–O–H), while that of plasma-treated c-NFs samples corresponded to ether-type compounds (C–O–C).

**Figure 11:**
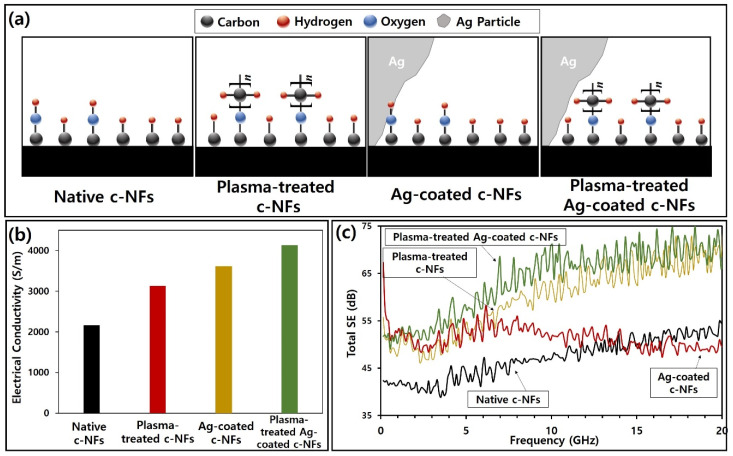
(**a**) Schematic revealing the surface chemical compositions of native c-NFs, plasma-treated c-NFs, Ag-coated c-NFs, and plasma-treated Ag-coated c-NFs; (**b**) bar graphs showing electrical conductivities of each sample; (**c**) variations in total SE as a function of operating frequency for each sample.

The combined results of Figure 10 and Table 4 indicate a more significant increase in the carbon component than in the oxygen component on the surface of plasma-treated c-NFs carbon fibers. Furthermore, the difference in binding energies observed for native c-NFs and plasma-treated c-NFs may imply a transformation of carbon–oxygen bonds from alcohol-type (C–O–H) to ether-type (C–O–C). Therefore, we suggest that this transformation caused an increase in the carbon component present on the surface of the carbon fibers. Furthermore, the transformation of O–H bonds in C–O–H groups into O–C bonds in C–O–C groups may have enhanced the magnetic characteristics of the surface of the plasma-treated carbon fibers. In other words, the increased number of electrons provided by the carbon atoms in the O–C bonds as compared with the hydrogen atoms contained by O–H bonds may have led to enhanced magnetic characteristics. Figure 11a provides a schematic of the surface chemical compositions of each sample. Figure 11b depicts the electrical conductivity of the samples, and Figure 11c shows the total SE spectra of the samples.

The observed total SE of plasma-treated c-NF was greater than 45 dB across the entire range of operating frequencies studied in this work. When compared with SE values previously reported in the literature for other materials, the SE values reported in the present study rank among the highest (see Table 5). These results suggest that H_2_ plasma-treated c-NFs can be used to manufacture effective electromagnetic wave shielding materials for use in diverse industrial fields.

## 4. Conclusions

Despite the surface electrical conductivity of H_2_ plasma-treated c-NFs being lower than that of Ag-coated c-NFs, H_2_ plasma treatment dramatically enhanced electromagnetic wave SE by enhancing absorption loss effect at high operating frequencies between 7.0 and 20.0 GHz. After 5 min of H_2_ plasma treatment, the proportion of carbon on the surface of the carbon fibers appeared to have increased via the transformation of alcohol-type (C–O–H) compounds into ether-type (C–O–C) compounds. Because more electrons were present in the carbon of O–C bonds than in the hydrogen of O–H bonds, this transformation may have enhanced the magnetic characteristics of the carbon fiber surface. The increased magnetic permeability led to a decrease in skin depth, which in turn increased the total SE.

## Figures and Tables

**Table 1:** Experimental conditions for H_2_ plasma treatment using the microwave plasma-enhanced chemical vapor deposition (MPECVD) system.

Substrate Temperature (°C)	H_2_ Gas Pressure (Torr)	H_2_ Gas Flow Rate (sccm)	H_2_ Plasma Reaction Time (min)	Microwave Power (W)
750	40	100	5	900

**Table 2:** Experimental conditions for producing Ag-coated carbon-based nonwoven fabrics (c-NFs) using a direct current (DC) sputtering system.

Ar Gas Pressure (Torr)	Ar Gas Flow Rate (sccm)	Total Reaction Time (min)	DC Power (kW)	Sputter Target
40	70	6.0	1.0	Ag

**Table 3:** Resistivity and electrical conductivity values of the studied samples.

Samples	Thickness (mm)	Resistivity *ρ* (Ω∙m)	Conductivity *σ* (S/m)	* Correction Factor *F* (w/s)
Native c-NFs	1.5	4.60 × 10^−4^	2.17 × 10^3^	0.99
c-NFs treated with H_2_ plasma for 5 min		3.20 × 10^−4^	3.13 × 10^3^	
Ag-coated c-NFs		2.77 × 10^−4^	3.61 × 10^3^	
Ag-coated c-NFs treated with H_2_ plasma for 5 min		2.42 × 10^−4^	4.13 × 10^3^	

* The correction factor value comes from Table 3 of Smits’s work [18].

**Table 4:** X-ray photoelectron spectroscopy (XPS) characterization of native and plasma-treated c-NFs samples.

Samples	Binding Energy (eV)	* Binding Energy of Reference Compounds (eV)
	O (1s)	O (1s)
Native c-NFs	532.78	532.8 (C–O–H)
c-NFs treated with H_2_ plasma for 5 min	532.57	532.5 (C–O–C)

* The reference compound biding energy values were obtained from Kerber et al. [26] and Beamson et al. [27].

**Table 5:** Previously reported shielding effectiveness (SE) values of carbon-based materials.

Carbon-Based Materials	Thickness (mm)	Conductivity or Sheet Resistance	Operating Frequency (GHz)	SE (dB)	Refs.
*CFC/*CNTs/*CIPs	6.0	-	1–18	52–73	[28]
25 wt% *MWCNTs/*PMMA	0.1	>10 S/cm	0.1–14	17–22	[29]
Activated carbon fiber/*PA	4.0	-	2–18	10–27	[30]
*CNTs-Ni_40_Co_60_/*PVDF	4.5	1.7 × 10^−4^ S/cm		11–41	[31]
Functionalized (maleic anhydride modified) *MWCNTs/*PMMA	1.0	1.33 × 10^6^ Ω/sq		13–18	[32]
33 wt% *GS/*PANI	2.4	20 S/cm		20–34	[33]
25 wt% *SWCNT/*PANI	2.4	34 S/cm		17–32	
*MG/*LDPE	2.0–2.1	-		5–21	[34]
*MWCNTs/*PCL	20.0	>4.0 S/m	0.04–40	60–80	[35]
Native c-NFs	1.5	2.17 × 10^3^ S/m	0.04–20	39–55	This Work
c-NFs treated with H_2_ plasma for 5 min	1.5	3.13 × 10^3^ S/m		46–75	
Ag coated c-NFs	1.5	3.61 × 10^3^ S/m		47–67	
Ag coated c-NFs plasma-treated with H_2_ plasma for 5 min	1.5	4.13 × 10^3^ S/m		49–75	

*CFC: layered carbon fiber composites, *CIPs: carbonyl iron powders, *CNTs: carbon nanotubes, *GS: graphene sheet, *LDPE: low density polyethylene, *MG: natural microcrystalline graphite, *MWCNTs: multi-walled carbon nanotubes, *PA: polyamide, *PANI: polyaniline, *PCL: polycaprolactone, *PMMA: poly(methyl methacrylate), *PVDF: polyvinylidene fluoride, *SWCNT: single-wall carbon nanotube.
